# Supplementary material for: Extracellular Vesicles in Liquid Biopsies as Biomarkers for Solid Tumors
Source: Cancers (Basel). 2023 Feb 18;15(4):1307. doi: 10.3390/cancers15041307 (PMC9953862; doi:10.3390/cancers15041307)
Supplement: Supplementary file 1 [file cancers-15-01307-s001.zip › cancers-2201101-SI.pdf]

**Supplementary Table S1.** Overview of circulating EV-associated biomarkers for selected solid tumor entities tested in a clinical setting. Listed are protein-, RNA-, and DNA-based biomarkers that were clinically assessed in patient cohorts suffering from glioblastoma multiforme (GBM), head and neck cancer (HNC), lung cancer (LC), breast cancer (BC), hepatocellular carcinoma (HCC), pancreatic ductal adenocarcinoma (PDAC), colorectal carcinoma (CRC), prostate cancer (PC) and osteosarcoma (OS).

| Type | Cancer entity | Biomarker | Clinical impact | Reference |
|---|---|---|---|---|
| Protein-based biomarkers | GBM | CD63, EGFR, EGFRvIII | Decrease after chemotherapy | Shao et al., 2012 [121] |
| | PDAC | CKAP4 | Decrease after resection | Kimura et al., 2019 [119] |
| | | EpCAM | High levels predict worse survival | Giampieri et al., 2019 [124] |
| | CRC | CD147 | Decrease after resection | Yoshioka et al., 2014 [116] |
| | | EGFR | High levels predict worse survival | Menck et al., 2017 [23] |
| | | GPC1 | Decrease after resection | Li et al., 2017 [118] |
| | | CPNE3 | High levels predict worse survival, diagnostic relevance | Sun et al., 2019 [95] |
| | | Hsp60 | Decrease after resection | Campanella et al., 2015 [117] |
| | LC | MUC1 | High levels predict worse survival | Menck et al., 2017 [23] |
| | | EGFR | Increased levels in patients | Yamashita et al., 2013 [94] |
| | | 19 proteins | Increased levels in patients, diagnostic relevance | Hoshino et al., 2020 [98] |
| | PC | PSMA | Decrease after prostatectomy | Biggs et al., 2016 [120] |
| | | PSA | Increased levels in patients, diagnostic relevance | Logozzi et al., 2019 [100] |
| | | Survivin | Increased levels in patients | Khan et al., 2012 [101] |
| | | 51 proteins | Increased levels in patients, diagnostic relevance | Hoshino et al., 2020 [98] |
| | BC | Del-1 | Decrease after resection | Moon et al., 2016 [102] |
| | | Survivin | Increased levels in patients, diagnostic relevance | Khan et al., 2014 [93] |
| | | CD82 | Expression correlates with disease progression | Wang et al., 2019 [97] |
| | | MUC1, EpCAM | High levels predict worse survival | Menck et al., 2017 [23] |
| | HNC | MUC1, EpCAM | High levels predict worse survival | Menck et al., 2017 [23] |
| | | CD3, PDL1 ($T_{reg}$ -derived | Increase in patients with recurrence | Theodoraki et al., 2019 [82] |
| | HCC | miR-1247-3p | High levels associated with lung metastasis | Fang et al., 2018 [136] |

| | | | | |
|---|---|---|---|---|
| RNA-based biomarkers | | miR-638 | Low levels associated with shortend survival | Shi et al., 2018 [138] |
| | PDAC | lncRNA HULC | Increased in patients | Takahashi et al., 2020 [103] |
| | | miR-21 | High levels associated with shortend survival | Goto et al., 2018 [104] |
| | CRC | ΔNp73 mRNA | High levels associated with shortend survival | Soldevilla et al., 2014 [96] |
| | | lncRNA CRNDE-p | Decrease after chemotherapy | Yu et al., 2017 [139] |
| | | miR-217 | Increase after chemotherapy | Yu et al., 2017 [139] |
| | | miR-17-5p, miR-92a-3p | High levels associated with disease progression | Fu et al., 2018 [137] |
| | LC | miR-23b-3p, miR-10b-5p, miR-21-5p | High levels associated with shortend survival | Liu et al., 2017 [105] |
| | | miR-425–3p | High levels predict worse survival after chemotherapy | Yuwen et al., 2019 [130] |
| | | miR-146a-5p | Low levels predict recurrence after chemotherapy | Yuwen et al., 2017 [134] |
| | | hsa-miR-320b, -c, -d | High levels in non-responders to anti-PD-1 therapy | Peng et al., 2020 [128] |
| | | PD-L1 mRNA | High levels in responders to anti-PD-1 therapy | Del Re et al., 2018 [127] |
| | PC | hsa-let-7a-5p | Increase after radiation therapy | Malla et al., 2018 [80] |
| | | miR-654-3p, miR379-5p | Predict efficacy of radiotherapy | Yu et al., 2018 [133] |
| | BC | miR-301, miR-155 | Decrease after neoadjuvant chemotherapy | Stevic et al., 2018 [122] |
| | | miR-21 | High levels correlate with tumor size and CTC concentration | Rodriguez-Martinez et al., 2019 [106] |
| | | lncRNA HOTAIR | Decrease after resection | Tang et al., 2019 [123] |
| | OS | Transcriptomic profile | Metastatic signature | Bao et al., 2018 [135] |
| | | RNA mutation burden | Increase upon metastasis | Bao et al., 2018 [135] |
| DNA-based biomarkers | PDAC | KRAS mutation | High detection rates correlate with disease progression and survival | Allenson et al., 2017 [107] |
| | GBM | IDH1 mutation | Decreased detection in low-grade gliomas | Garcia-Romero et al., 2017 [140] |
| | LC | EGFR mutation | High detection efficiency when combined with cfDNA | Castellanos-Rizaldos et al., 2019 [112] |
| | CRC | Several mutations | Detection rates correlate with tumor size | Thakur et al., 2021 [109] |
